# Severe anemia in patients with Propionic acidemia is associated with branched-chain amino acid imbalance

**DOI:** 10.1186/s13023-021-01865-7

**Published:** 2021-05-18

**Authors:** Sinziana Stanescu, Amaya Belanger-Quintana, Borja Manuel Fernandez-Felix, Francisco Arrieta, Victor Quintero, Maria Soledad Maldonado, Patricia Alcaide, Mercedes Martínez-Pardo

**Affiliations:** 1 Servicio de Pediatria, Unidad de Enfermedades Metabólicas, Hospital Universitario Ramón y Cajal, IRYCIS, Crta de Colmenar Viejo, km 9,100, 28034 Madrid, Spain; 2 Unidad de Bioestadistica Clinica, Instituto Ramon y Cajal de Investigacion Sanitaria, CIBER Epidemiología y Salud Pública (CIBERESP), Hospital Universitario Ramón y Cajal, Crta de Colmenar Viejo, km 9,100, 28034 Madrid, Spain; 3 Unidad de Enfermedades Metabólicas, Hospital Universitario Ramón y Cajal, IRYCIS, CIBER-OBN, Crta de Colmenar Viejo, km 9,100, 28034 Madrid, Spain; 4 Unidad de Oncohematologia, Servicio de Pediatria, Hospital Universitario Ramón y Cajal, IRYCIS, Crta de Colmenar Viejo, km 9,100, 28034 Madrid, Spain; 5 Centro de Diagnóstico de Enfermedades Moleculares, Centro de Biología Molecular, Universidad Autónoma de Madrid, CIBERER, IdiPAZ, C/Francisco Tomás y Valiente, 7, 28049 Madrid, Spain

**Keywords:** Propionic acidemia, Anemia, Diet, Protein-restricted, Branched-chain amino acids imbalance

## Abstract

**Background:**

Propionic acidemia (PA), an inborn error of metabolism, is caused by a deficiency in propionyl-CoA carboxylase. Patients have to follow a diet restricted in the propiogenic amino acids isoleucine (Ile), valine (Val), methionine (Met) and threonine (Thr); proper adherence can prevent and treat acute decompensation and increase life expectancy. However, chronic complications occur in several organs even though metabolic control may be largely maintained. Bone marrow aplasia and anemia are among the more common.

**Materials and methods:**

In this retrospective study, data for patients with PA being monitored at the *Hospital Ramón y Cajal* (Madrid, Spain) (n = 10) in the past 10 years were examined to statistically detect relationships between persistent severe anemia outside of metabolic decompensation episodes and dietary practices such as natural protein intake and medical food consumption (special mixture of precursor-free amino acids) along with plasma levels of branched-chain amino acids (BCAA). High ferritin levels were deemed to indicate that a patient had received repeated transfusions for persistent anemia since data on hemoglobin levels at the moment of transfusion were not always passed on by the attending centers.

**Results:**

Three patients had severe, persistent anemia that required repeated blood transfusions. Higher medical food consumption and plasma Leu levels were associated with iron overload. Notably, natural protein intake and plasma Val were negatively correlated with ferritin levels. We also observed an inverse relationship between plasma Val/Leu and Ile/Leu ratios and ferritin.

**Conclusion:**

The present results suggest that severe anemia in patients with PA might be associated with low natural protein intake and BCAA imbalance.

**Supplementary Information:**

The online version contains supplementary material available at 10.1186/s13023-021-01865-7.

## Background

Propionic acidemia (PA; OMIM 606054) is a rare, inherited (autosomic recessive) metabolic disease caused by a deficiency in propionyl-CoA carboxylase, a mitochondrial enzyme that transforms propionyl-CoA into methylmalonyl-CoA. Propionyl-CoA is an intermediate in the metabolism of the amino acids isoleucine (Ile), valine (Val), methionine (Met) and threonine (Thr). Other sources of propionyl-CoA include odd-numbered long-chain fatty acids (OLCFAs), cholesterol, and propionic acid generated by gut bacteria. Propionyl-CoA deficiency leads to the accumulation of 3-hydroxy propionic acid and methyl citrate, propionyl carnitine and tiglyl glycine among other abnormal intermediates of propionyl-CoA metabolism [1].

Commonly beginning in neonatal life, patients with PA experience acute metabolic decompensation during scenarios involving increased catabolism, e.g., infections or prolonged fasting. These episodes are the result of intoxication by alternative metabolism products, leading to lactic acidosis, ketosis, hyperammonemia, and multiorgan failure [2]. Moreover, patients with PA, even those with good metabolic control, suffer chronic complications involving organs with high energy demands, e.g., the central nervous system (encephalopathy, abnormal movements, epilepsy, psychomotor delay, ataxia, lesions in the basal ganglia similar to those seen in Leigh's syndrome, and atrophy of the optic nerve), the heart (dilated cardiomyopathy, arrhythmias), bone marrow (bone marrow aplasia, cytopenia), and the gastrointestinal tract (pancreatitis, hepatitis), etc [3]. No complete pathophysiological explanation for this is yet available. Treatment is based largely on dietary natural protein restriction to limit the intake of amino acids providing precursors of propionyl-CoA (Met, Thr, Val and Ile), together with a special mixture of precursor-free amino acids (SMAA) when the natural protein tolerance is below FAO/WHO/UNU (2007) recommendations, plus the administration of carnitine and metronizadole [2, 4].

The main aim of the present work was to detect the possible relationships between severe anemia in patients with PA outside of metabolic decompensation episodes and nutritional parameters, such as protein intake and BCAA plasma levels.

## Materials and methods

The medical records of patients with PA being monitored in the past 10 years at the Metabolic Disease Unit, *Hospital de Ramón y Cajal* (Madrid, Spain) (N = 10), were examined in order to identify those with significant anemia outside of metabolic decompensation episodes. Since these patients receive periodic transfusions of blood derivatives at hospitals other than the above, high plasma ferritin was used as a marker of iron overload secondary to repeated transfusions for severe anemia. This proxy was employed since hemoglobin levels at the moment of transfusion were not available for all patients (they were not passed on by all attending centers). The blood transfusion was provided by their attending hospitals when hemoglobin concentrations were below 7.5 g/dl; genetic causes of hyperferritinemia (e.g. haemochromatosis) and renal disease were discarded. Relationships were sought between plasma ferritin, the natural protein intake (NPI), SMAA consumption and the BCAA (Ile, Val, Leu, Val/Leu and Ile/Leu ratios) plasma levels. Periods of metabolic decompensation were not taken into account in analysis since severe pancytopenia can occur at such times. Infection episodes were also excluded given the possible interference with plasma ferritin concentrations. Blood and plasma samples were collected with 4–6 h of fasting. When prescribing total protein, the WHO/FAO/UNU (2007) safe levels of protein intake were used as point of reference [2].

Amino acids in plasma/serum were analyzed by ion-exchange chromatography with ninhydrine. All lab measurements were completed in our ERNDIM approved, reference laboratory (CEDEM, Centro de Diagnostico de Enfermedades Moleculares, Universidad Autonoma, Madrid).

### Statistical analysis

We fitted a multilevel linear regression model with plasma ferritin as the dependent continuous outcome and each amino acid together with natural protein and SMAA intakes as the independent variable. We defined a two-level model for measures (first level) within patients (second level). Thus, we considered repeated measures were made for each patient. We considered statistically significant for a p-value < 0.05. All analyses were performed using Stata software version 16 [5].

## Results

Data of ten PA patients (aged 5–38 years) were examined. The number of measures for each patient ranged from 3 to 24, see Table 2 and Additional file 1. Table 1 shows the demographic and clinical characteristics of the patients. 3/10 patients showing persistent severe anemia and requiring repeated transfusions were detected (patients 1, 2 and 3). All three were diagnosed when neonates, and at the time of study they all showed multisystem disease with different chronic complications, see Table 1. The iron overload caused by these transfusions led all three to require iron chelation treatment with deferasirox (Exjade^®^). Although the renal function estimated by plasma creatinine was normal (see Additional file 2), treatment with erythropoietin was prescribed in two patients (patient 1 and 2), but without benefit.

**Table 1:** Demographic and clinical data of 10 PA patients included in the study

	Sex	Current age (years)	Age at diagnosis	Genetics	Clinical course (long term complications)
1	F	32	Neonatal	PCCB gene p.Gly407Argfs*14/p.Arg165Gln	Peripheric neuropathy, neuromotor delay, pancreatitis, thrombopenia Severe persistent anemia
2	M	12	Neonatal	PCCB gene p.Gly407Argfs*14/ p. Arg410Trp	Severe neuromotor delay, leukopenia Severe persistent anemia
3	M	Died (5)	Neonatal	PCCA gene p.Leu470Arg/p.Leu470Arg	Severe neuromotor delay Choreoathetosis, basal ganglia involvement, leukopenia, frequent infections, dilated cardiomyopathy, pancreatitis Severe persistent anemia
4	F	15	4 months	PCCA gene p.Gly477fs*9/p.Cys616_Val633del	Pancreatitis
5	M	9	Neonatal screening	PCCB p.Asn536Asp/p.Asn536Asp	Autism
6	F	27	4 months	PCCB gene p.Gly407Argfs*14/p.Glu168Lys	–
7	M	12	6 months	PCCA gene p.Gly477fs*9/p.Cys616_Val633del	Sever neuromotor delay
8	F	13	6 months	PCCB gene p.Arg512Cys/p.Gly255Ser	Neuromotor delay, epilepsy, pancreatitis, myositis
9	F	38	Neonatal	PCCB p.Glu168Lys/p.? (c.183 + 3G > C)	Neuromotor delay
1	F	32	Neonatal	PCCB gene p.Gly407Argfs*14/p.Gly407Argfs*14	Neuromotor delay, dilated cardiomyopathy

Median natural protein prescription was below the WHO/FAO/UNU (2007) safe levels, therefor SMAA supplements was prescribed for the all the patients included (see Table 2). All received treatment with metronidazole, carnitine and Ile supplements; the caloric intake was maintained between 100 and 150 kcal/kg/day.

**Table 2:** Descriptive statistics for ferritin levels and nutritional support: natural protein intake (NPI) (g/kg/day), special mixture of precursor-free amino acids (SMAA) intake (g/kg/day), total protein intake, % of protein provided by natural protein intake

Patient	1	2	3	4	5	6	7	8	9	10
Measures (N)	N = 10	N = 24	N = 9	N = 4	N = 12	N = 5	N = 9	N = 3	N = 3	N = 7
*Natural protein intake (NPI) (g/kg/day)*										
Mean (sd) Min; max	0.29 (0.14) 0.11; 0.46	0.55 (0.25) 0.17; 0.89	0.79 (0.29) 0.4; 1.2	0.58 (0.06) 0.5; 0.65	0.96 (0.07) 0.87; 1.1	0.39 (0.01) 0.38; 0.41	0.72 (0.06) 0.6; 0.3	0.24 (0.00) 0.24; 0.24	0.32 (0.09) 0.21; 0.36	0.28 (0.09) 0.16; 0.36
*SMAA intake (g/kg/day)*										
Mean (sd) Min; max	1.23 (0.23) 0.92; 1.5	1.97 (0.33) 1.61; 2.77	1.58 (0.2) 1.33; 1.93	1.41 (0.2) 1.2; 1.66	2 (0.09) 1.86; 2.15	0.62 (0.02) 0.61; 0.66	2.3 (0.13) 2.1; 2.5	1.7 (0.00) 1.7; 1.7	0.96 (0.09) 0.91; 1.07	1.0 (0.00) 1.0; 1.0
*Amount of total protein from natural protein (%)*										
Mean (sd) Min; max	19.1 (8.5) 7.2; 28.5	22 (9.5) 7.4; 35	33 (10) 17; 44	29.5 (4.9) 23; 33.3	35.5 (1.8) 30; 36	38.5 (0.00) 38.5; 38.5	24 (1.9) 19; 26	12 (0.00) 12; 12	25 (7) 16.6; 29	21.5 (5.8) 14; 26
*Ferritin (ng/ml). NV: 20–250 ng/ml*										
Mean (sd) Min; max	429.1 (223.6) 124; 829	873.1 (768.1) 142; 2926	556.2 (657.2) 103; 2085	56 (28.3) 24; 93	24.6 (11.2) 9; 50	34.4 (6.6) 28; 44	76 (39) 25; 139	25 (23) 10; 53	72.8 (27.3) 51; 103	245 (186) 111; 651

**Fig. 1:**
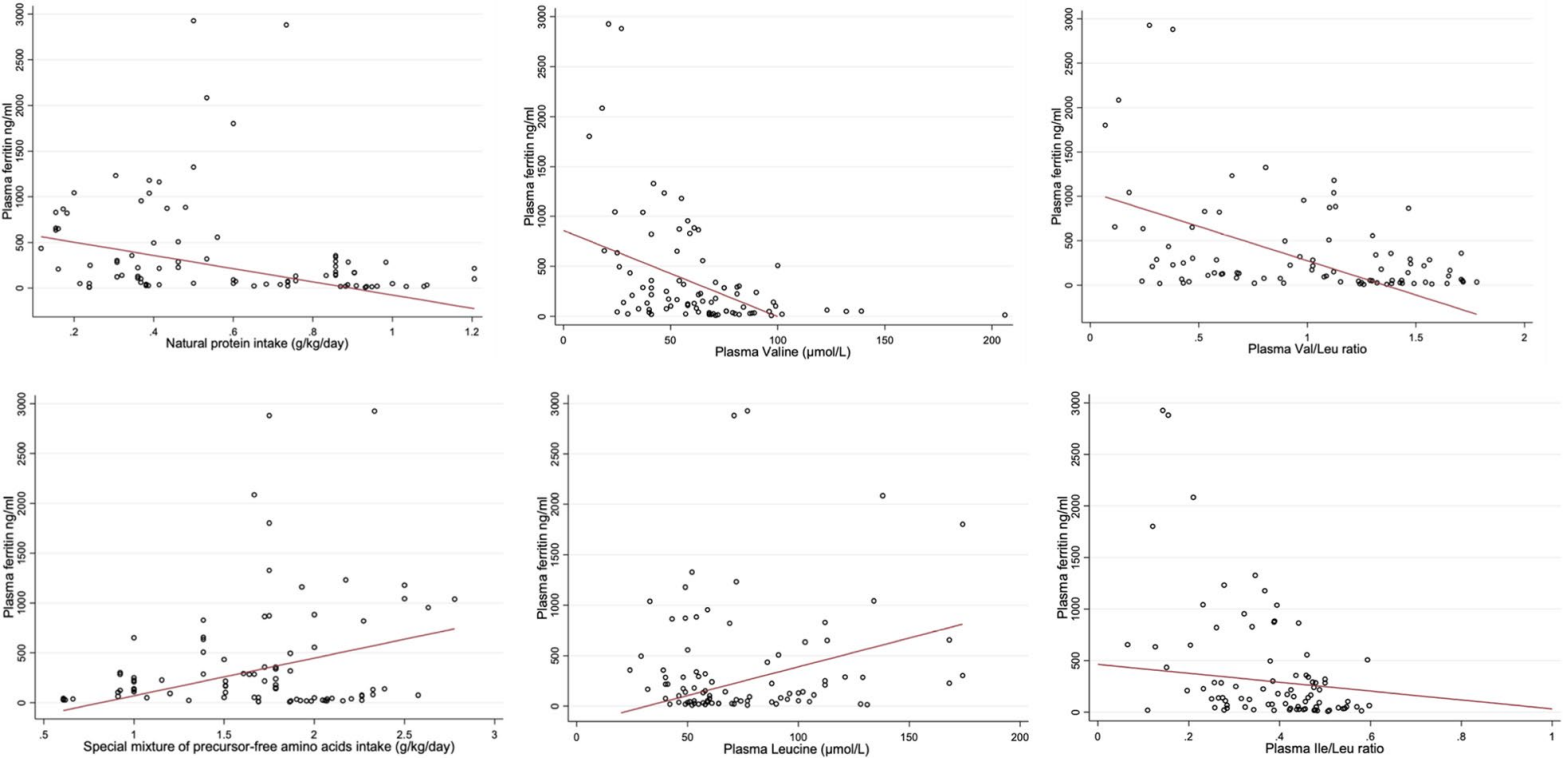
Correlation between natural protein intake, SMAA consumption and plasma BCAA with respect to plasma ferritin. Scatter plot with the values of the 10 patients; the red lines represent the predictions of the multilevel model. Val: Valine; Ile: Isoleucine; Leu: Leucine

The natural protein intake (g/kg/day) was negatively associated with iron overload (p-value 0.003, regression coefficient (95% C.I): − 72.1 (− 119.6; − 24.5)), whereas the SMAA consumption (g/kg/day) resulted in higher plasma ferritin levels (p-value 0.019, regression coefficient (95% C.I): 37.8 (6.1; 69.5)). Notably, the hyperferritinemia was associated with lower levels of Val (p-value < 0.001, regression coefficient (95% C.I): − 8.6 (− 12.3; − 4.8)) and higher levels of Leu (p-value 0.001, regression coefficient (95% C.I): 5.6 (2.2; 9.1), see Fig. 1 and Table 3. Also, a negative correlation was observed between the ratios of Val/Leu (p-value < 0.001, regression coefficient (95% C.I): − 771.5 (− 987.4; − 555.5)) and Ile/Leu (p-value < 0.018, regression coefficient (95% C.I): − 431 (− 788.6; − 73.3)) with the ferritin suggesting that BCAA imbalance was potentially detrimental for the bone marrow, see Fig. 1 and Table 3.

**Table 3:** Analysis of plasma amino acid plasma levels and dietary practices (natural protein and special amino acid mixture intake) according to ferritin levels using multilevel linear regression

	Regression coefficient (95% CI)	*p value*
Natural protein intake (g/kg/day)	− 72.1 (− 119.6; − 24.5)	0.003
Special mixture of precursor-free amino acids intake (g/kg/day)	37.8 (6.1; 69.5)	0.019
Valine (µmol/L)	− 8.6 (− 12.3; − 4.8)	< 0.001
Isoleucine (µmol/L)	− 5.2 (− 12; 1.5)	0.128
Leucine (µmol/L)	5.6 (2.2; 9.1)	0.001
Valine/Leucine	− 771.5 (− 987.4; − 555.5)	< 0.001
Isoleucine/Leucine	− 431 (− 788.6; − 73.3)	0.018

## Discussion

The long-term complications suffered by patients with PA affects their prognosis [3]. Certainly, they increase morbidity and mortality rates outside of the acute decompensation episodes. Given the small number of patients with PA, the actual prevalence of hematological complications is unknown. A recent meta-analysis determined anemia to be more common than cardiomyopathy or lesions in the basal ganglia [3]. In another study of the long-term complications of organic acidemia and urea cycle disorders (as recorded in the European Registry of Organic Acidemia and Urea Cycle Disorders), the prevalence of anemia among patients with PA was determined to be 22%, while figures for leucopenia and thrombocytopenia reached 18% [6]. Pancytopenia has generally been described as occurring during periods of acute decompensation, but patients can also experience anemia, neutropenia or thrombocytopenia outside of these times [2]. The reason for these hematological problems may lie in the toxicity of accumulating metabolites such as 3-hydroxy propionic acid, methyl citrate or tiglyl glycine, a lack of certain nutrients [6], or mitochondrial dysfunction [3]. The pancytopenia seen during decompensation episodes is generally reversible [2, 7, 8], supporting the idea that accumulating toxic metabolites are to blame. However, why hematological problems should occur during times of metabolic stability remains unclear.

The blood is a tissue with a high regeneration rate. The need for nutrients—especially amino acids—is therefore high compared to other tissues, leaving the hematopoietic tissue sensitive to malnutrition [9]. Certainly, anemia associated with protein-energy malnutrition is relatively common in children and elderly people in general [10]. The constant production of blood cells from hematopoietic stem cells (HSC) is also influenced by the latter’s microenvironment, a complex biological niche [11]. In recent years, interest has grown in understanding the influence of diet on the physiology and viability of HSC. Several studies, particularly those investigating the pre-transplant conditioning of the bone marrow, have shown the influence exerted by dietary BCAAs and cysteine (Cys) on the function of HSC and their microenvironment. Indeed, Val and Cys have been reported indispensable for the maintenance of HSC [12]. In vitro, neither human nor rat HSC can proliferate in media without these amino acids, and rats fed a Val-restricted diet show a reduced HSC count within a week [12]. In another study that examined the effect of BCAA balance on HSC viability, restricting the Val intake by 10% led to a significant fall in HSC numbers. The same 10% restriction in Val in the presence of increased Ile and Leu led to the complete blockage of HSC proliferation [13]. The reason why HSC are so sensitive to a reduced Val intake and to disequilibrium between BCAAs has not been explored. However, it may involve Val's role as a structural unit of proteins, or some relationship with cell signaling [11].

The above evidence suggests that an imbalance of the BCAA can have a negative effect on the bone marrow. Certainly, among the present patients, reduced plasma Val negatively correlated with ferritin levels, whereas Leu was directly associated with iron overload. Moreover, there was an inverse relation between the ratios Val/Leu and Ile/Leu with plasma ferritin.

The dietary recommendations for PA patients are based on the restriction of natural protein intake, the use of SMAA together with the avoidance of prolonged fasting and adequate energy supply [2]. The SMAA contains no Val, Ile, Met and Thr, but a normal-high Leu levels and they are recommended if natural protein tolerance is below FAO/WHO/UNU safe levels [2]. The actual amount of medical food versus intact protein intakes are not detailed by the current guidelines and protein tolerance should be titrated individually [2, 14].

Thus, in PA patients, the combination between the natural protein restriction and the use of SMAA might result in an imbalanced BCAA dietary content with high Leu/Val or Leu/Ile ratios [15] that will directly reflect in BCAA plasma levels [16, 17], since these are essential amino acid entirely provided by the diet. Still, there are scarce studies investigating the impact of an BCAA imbalanced diet in organic aciduria patients. In an observational study in methyl malonic acidemia patients, increased leucine intake was associated with adverse growth outcomes [16].

In mice, elevated Leu was found to reduce plasma Val and Ile via system L amino acid transporter [18]. Animals fed high doses of Leu show low central nervous system concentrations of tryptophan, Val, Ile, Met and alanine, which might affect the synthesis of neurotransmitters [19]. Other authors have reported the importance of BCAA equilibrium in body growth and cellular immunity in animal models [20, 21]. In normal adults, high Leu intake was found to increase ammonia levels and to significantly decrease plasma concentration of Val and Ile [22].

The present work is the first to provide positive evidence of a nutritional component underlying the hematological complications of PA during stable metabolic periods. Our findings suggest that the natural protein and medical food intake as well as the balance of BCAA plasma levels might play an important role in the onset and development of severe anemia in PA. The natural protein restriction below 25–30% of total protein intake together with generous use of medical food might result in branched-chain amino acids imbalance and should be avoided.

The small number of patients examined is an important limitation of this study. Confirmatory studies involving international registries should be performed.

## Conclusion

Patients with PA can experience serious hematological complications even during periods of metabolic stability. The severe anemia may be due to an imbalance of BCAA plasma levels, presumably due to a low natural protein intake/high synthetic protein consumption. Further work is needed to confirm the importance of dietary practices and the BCAA equilibrium in the development of long-term hematological complications in patients with PA.

## Supplementary Information

**Additional file 1.** Descriptive analysis of plasma branched-chain amino acids levels. Val: valine; Ile: isoleucine; Leu: Leucine.

**Additional file 2.** Excell data.

## Data Availability

All data generated or analyzed during this study are included in this published article and its supplementary information files.

## Abbreviations

PA: Propionic academia
BCAA: Branched-chain amino acids
Val: Valine
Ile: Isoleucine
Leu: Leucine
Met: Methionine
Thr: Threonine
OLCFAs: Odd-numbered long-chain fatty acids
SMAA: Special mixture of precursor-free amino acids
NPI: Natural protein intake
