# Supplementary material for: Severe anemia in patients with Propionic acidemia is associated with branched-chain amino acid imbalance
Source: Orphanet J Rare Dis. 2021 May 18;16:226. doi: 10.1186/s13023-021-01865-7 (PMC8130149; doi:10.1186/s13023-021-01865-7)
Supplement: Supplementary file 1 — Additional file 1. Descriptive analysis of plasma branched-chain amino acids levels. Val: valine; Ile: isoleucine; Leu: Leucine. [file 13023_2021_1865_MOESM1_ESM.docx]

Supplementary material. Descriptive analysis of plasma BCAA levels

| Patient | 1 | 2 | 3 | 4 | 5 | 6 | 7 | 8 | 9 | 10 |
| --- | --- | --- | --- | --- | --- | --- | --- | --- | --- | --- |
| Measures (N) | N = 10 | N = 24 | N = 9 | N = 4 | N = 12 | N = 5 | N = 9 | N = 3 | N = 3 | N = 7 |
| Val (µmol/L). NV: 212±53 µmol/L | | | | | | | | | | |
| Mean (sd)  Min; max | 55 (26.5)  19 ; 100 | 53.1 (21.5)  12 ; 98 | 41.2 (12.9)  18 ; 56 | 73 (11.5)  57 ; 84 | 77.7 (17)  41 ; 102 | 77.2 (11.2)  63 ; 88 | 38.5 (11)  25 ; 62 | 138 (67.5)  71 ; 206 | 118 (17)  99 ; 132 | 57.4 (15.5)  32 ; 81 |
| Ile (µmol/L). NV: 53±16 µmol/L | | | | | | | | | | |
| Mean (sd)  Min; max | 33.3 (18.1)  11 ; 67 | 20.1 (5.5)  10 ; 31 | 18.3 (6.6)  7 ; 29 | 30 (5.4)  26 ; 38 | 25.5 (6.6)  14 ; 39 | 26.6 (1.1)  25 ; 28 | 23 (7.5)  13 ; 38 | 97 (53.6)  57 ; 158 | 37.6 (5)  33 ; 43 | 57.4 (15.5)  32 ; 81 |
| Leu (µmol/L). NV: 108±31 µmol/L | | | | | | | | | | |
| Mean (sd)  Min; max | 124.8 (33.9)  86 ; 174 | 62.5 (29.4)  24 ; 174 | 48.2 (31.4)  20 ; 138 | 63.5 (10.6)  53 ; 78 | 66 (24)  42 ; 128 | 58.4 (5)  51 ; 64 | 73 (23)  40 ; 105 | 94 (40)  52 ; 131 | 70 (8.7)  60 ; 77 | 105 (9)  88 ; 113 |
| Val/Leu | | | | | | | | | | |
| Mean (sd)  Min; max | 0.46 (0.27)  0.11 ; 1.09 | 1.01 (0.48)  0.06 ; 1.7 | 1.05 (0.37)  0.13 ; 1.65 | 1.17 (0.24)  0.9 ; 1.43 | 1.3 (0.41)  0.32 ; 1.78 | 1.3 (0.16)  1.15 ; 1.54 | 0.5 (0.2)  0.23 ; 0.87 | 1.44 (0.11)  1.36 ; 1.57 | 1.69 (0.03)  1.65 ; 1.71 | 0.56 (0.2)  0.28 ; 0.92 |
| Ile/Leu | | | | | | | | | | |
| Mean (sd)  Min; max | 0.27 (0.15)  0.06 ; 0.59 | 0.36 (0.12)  0.12 ; 0.6 | 0.41 (0.08)  0.21 ; 0.5 | 0.47 (0.06)  0.42 ; 0.54 | 0.42 (0.13)  0.11 ; 0.54 | 0.45 (0.04)  0.42 ; 0.52 | 0.32 (0.05)  0.25 ; 0.41 | 1.4 (1.4)  0.57 ; 3.04 | 0.53 (0.05)  0.48 ; 0.59 | 0.26 (0.05)  0.19 ; 0.36 |
